# Randomised trial of glutamine and selenium supplemented parenteral nutrition for critically ill patients. Protocol Version 9, 19 February 2007 known as SIGNET (Scottish Intensive care Glutamine or seleNium Evaluative Trial)

**DOI:** 10.1186/1745-6215-8-25

**Published:** 2007-09-20

**Authors:** Peter JD Andrews, Alison Avenell, David W Noble, Marion K Campbell, Claire G Battison, Bernard L Croal, William G Simpson, John Norrie, Luke D Vale, Jonathon Cook, Robyn de Verteuil, Anne C Milne, The Trials Management Group

**Affiliations:** 1 Department of Anaesthesia, Critical Care & Pain Medicine, University of Edinburgh & Consultant, Critical Care, Western General Hospital Lothian University Hospitals Division, Edinburgh EH4 2XU, Scotland, UK; 2 Health Services Research Unit, Health Sciences Building, University of Aberdeen, Foresterhill, Aberdeen AB25 2ZD, Scotland, UK; 3 Health Economics Research Unit, University of Aberdeen, Polwarth Building, Foresterhill, Aberdeen AB25 2ZD, Scotland, UK; 4 Department of Anaesthetics & Intensive Care, Aberdeen Royal Infirmary, Foresterhill, Aberdeen AB25 2ZN Scotland, UK; 5 Department of Clinical Biochemistry, Aberdeen Royal Infirmary, Foresterhill, Aberdeen AB25 2ZN, Scotland, UK

## Abstract

**Background:**

Mortality rates in the Intensive Care Unit and subsequent hospital mortality rates in the UK remain high. Infections in Intensive Care are associated with a 2–3 times increased risk of death. It is thought that under conditions of severe metabolic stress glutamine becomes "conditionally essential". Selenium is an essential trace element that has antioxidant and anti-inflammatory properties. Approximately 23% of patients in Intensive Care require parenteral nutrition and glutamine and selenium are either absent or present in low amounts. Both glutamine and selenium have the potential to influence the immune system through independent biochemical pathways. Systematic reviews suggest that supplementing parenteral nutrition in critical illness with glutamine or selenium may reduce infections and mortality. Pilot data has shown that more than 50% of participants developed infections, typically resistant organisms. We are powered to show definitively whether supplementation of PN with either glutamine or selenium is effective at reducing new infections in critically ill patients.

**Methods/design:**

2 × 2 factorial, pragmatic, multicentre, double-blind, randomised controlled trial. The trial has an enrolment target of 500 patients. Inclusion criteria include: expected to be in critical care for at least 48 hours, aged 16 years or over, patients who require parenteral nutrition and are expected to have at least half their daily nutritional requirements given by that route.

Allocation is to one of four iso-caloric, iso-nitrogenous groups: glutamine, selenium, both glutamine & selenium or no additional glutamine or selenium. Trial supplementation is given for up to seven days on the Intensive Care Unit and subsequent wards if practicable. The primary outcomes are episodes of infection in the 14 days after starting trial nutrition and mortality. Secondary outcomes include antibiotic usage, length of hospital stay, quality of life and cost-effectiveness.

**Discussion:**

To date more than 285 patients have been recruited to the trial from 10 sites in Scotland. Recruitment is due to finish in August 2008 with a further six months follow up. We expect to report the results of the trial in summer 2009.

**Trial registration:**

This trial is registered with the International Standard Randomised Controlled Trial Number system. **ISRCTN87144826**

## Background

Intensive care unit (ICU) and subsequent hospital mortality in the UK remains high, with figures of 15–20% and 30% respectively [1]. The cost per ICU bed-day exceeds £1,100 based on a mean from all trusts in England and Wales [2]. In many countries, including the UK, provision of intensive care is inadequate to meet demand. Infections acquired on the ICU have been associated with a two to three fold increased risk of death [3], this is associated with both illness and drug related impairment of the patient's immune system. Multiple portals for infection include: tracheal tubes, nasogastric tubes, chest and abdominal drains, central venous, pulmonary arterial and urinary catheters, wounds, and infective loci present before admission to ICU. These infections increase mortality, morbidity, length-of-stay, antibiotic usage and costs of care. As a result 40–50% of patients on ICU are prescribed antibiotics at any one time [2]. Such concentrated antibiotic usage may contribute to ICU, hospital and even community antibiotic resistance, which is a major health care issue [2,4-6].

A European point prevalence study in ICUs showed that 66% of patients were receiving artificial nutritional support, 42% of whom were receiving either parenteral nutrition alone (23%) or parenteral combined with enteral nutrition (19%). [7].

Although the enteral feeding route is preferred to parenteral (reduced costs of administration and reduced risk of infective complications from the intravenous line), parenteral nutrition (PN) retains an important supportive role in the management of ICU patients, many of whom cannot be fed effectively by the enteral route because of gastro-intestinal dysfunction [8].

### Glutamine

Standard amino acid formulations do not contain any of the amino acid glutamine, due to pharmaceutical stability problems. In the past glutamine was considered a non-essential amino acid therefore its omission from parenteral nutrition preparations was not considered to be a problem, as it was thought that the body would synthesise its own. Glutamine has essential roles in acid-base balance, nitrogen transport and maintaining muscle mass and function, and is an energy source for rapidly dividing cells, particularly those of the immune system and gut. As the most abundant amino acid in the body, it is now recognised that glutamine synthesis and release is insufficient to meet demands under severe metabolic stress. Glutamine must therefore be supplied from nutritional sources if levels are to be maintained. Several studies have shown that glutamine levels decrease markedly after major surgery and during critical illness. Decreased serum glutamine has been associated with immune dysfunction in animal models and death or infectious complications in septic patients. In randomised controlled trials (RCTs) amongst patients, glutamine-supplemented PN has been associated with improved nitrogen balance, higher intra-muscular glutamine levels, improved mood, enhanced immune cell function and no elevation of proinflammatory cytokine profile [9-14]. In animal models glutamine enhances intracellular glutathione (which combats free radical tissue damage) [15]. Such effects of glutamine support on the stressed immune system in the patient receiving critical care could lead to '*successful inflammation'*, i.e. the ability to combat infection, and decrease the systemic inflammatory response syndrome, where excessive free radical induced damage occurs.

Thus provision of glutamine could be of considerable benefit in metabolically stressed critically ill patients. Although the proposed benefits are biologically plausible it is unclear whether they will be obtained in practice and there is a danger that glutamine-containing PN solutions will be incorporated into practice without adequate evaluation, thus our evaluation is timely.

### Selenium

Selenium is an essential trace element found in a group of proteins known as selenoproteins and which is present in only modest amounts within standard PN solutions. Selenium, through glutathione peroxidases and thioredoxin reductase is involved in anti-oxidant defences, and also has anti-inflammatory properties [16,17] mediated by inhibition of lipid peroxidation. Selenium is also essential to the production of thyroid hormones [18]. It is noteworthy that populations in many areas of Europe, including Scotland, are relatively selenium deficient [17]. Given the widely documented immunosuppression of critical illness, and its correlation with mortality and the purported roles of reactive oxygen species in sepsis, pancreatitis, organ dysfunction and other inflammatory conditions, it is biologically plausible that enhanced selenium supplementation could also be of benefit to the critically ill ICU patient requiring parenteral nutrition.

It can be seen from the above background that both glutamine and selenium have the potential to influence the immune system, through different sites in biochemical pathways. Thus a factorial design for the proposed RCT is appropriate to test the two hypotheses.

### Systematic reviews

A systematic review of glutamine supplementation in critically ill (including following major surgery) adults [19] found 14 small RCTs reporting data from 737 patients on mortality, infectious complications, and length of hospital stay. When both parenteral and enteral routes of supplementation are combined, complications were lower in the glutamine group (risk ratio 0.81, 95% CI 0.67 to 0.99). There was also a trend towards a reduction in mortality (risk ratio 0.79, 95% CI .59 to 1.04) and reduced length of hospital stay, especially for surgical patients.

A more recent systematic review using random effects methods of meta-analysis [20] found that parenteral glutamine in critical illness was associated with a relative risk for mortality of 0.75 (95% CI 0.52 to 1.07) and for infection of 0.71 (95% CI 0.49 to 1.05). There was however, evidence of publication bias in the reporting of participants' infections. The existing evidence is suggestive of a positive role for glutamine supplementation but a further large trial is required to determine whether any differences are statistically and clinically important.

A Cochrane systematic review of selenium supplementation in critical illness, has identified seven randomised controlled trials of sodium selenite supplementation in critical illness [21]. Five trials examined the effect of additional selenium supplementation on mortality at 28 days, and showed a reduction trend in those supplemented with selenium (relative risk 0.71, 95% CI 0.43 to 1.17, random effects model). The relative risk for mortality, whenever determined, for general intensive care patients was 0.75 (95% CI 0.59 to 0.96, random effects model). Based on reported information, these were poor quality trials. Indeed, only one clearly reported concealment of allocation and a further three trials undertook intention to treat analysis.

In a systematic review of antioxidant nutrients in critical illness, Heyland and colleagues [22] found that selenium supplementation (alone and in combination with other antioxidants) may be associated with reduced mortality (RR 0.59, 95% CI 0.32 to 1.08). Clearly, a larger trial to examine the clinical effects of additional selenium supplementation in this patient group is required.,

#### Pilot Study

The purpose of the pilot study was to test the full protocol with the aim of assessing feasibility and refining PN stock management. It was carried out in two Scottish centres; Aberdeen and Edinburgh. Patients enrolled to the pilot study were recruited and processed in the same manner as the full trial and we plan to analyse these data in the final data set.

## Methods and Design

### The questions which this protocol will address

#### The questions which this trial will address

• Does the inclusion of glutamine, additional selenium or the two in combination, in a standard preparation of PN improve the outcome for critically ill patients particularly in terms of participants with infections, mortality, ICU and acute hospital stay?

• Is supplementation of PN with glutamine, additional selenium, or the two in combination cost-effective?

### Trial recruitment

The trial will involve people expected to be on intensive care and high dependency units (levels 2 and 3 care) under the care of the intensive care unit consultants for at least 48 hours, aged 16 years or over, who require parenteral nutrition and are expected to have at least half of daily nutritional requirements given by that route. Pregnant women and people whose expected stay in the UK is less than six months will not be considered for the study. Patients with severe renal failure (creatinine clearance < 10 ml/min and not on renal replacement therapy) will also be excluded.

In the case of hepatic failure, metabolism by the liver is the main route of elimination for many drugs and nutrients, but the hepatic reserve appears to be large and liver disease has to be severe before important changes occur. Routine liver function tests (LFTs) are a poor guide to the capacity of the liver to metabolise drugs, and it is not possible to predict the extent to which the metabolism of a particular drug or nutrient may be impaired. Enrolment will depend on the decision of the local physician. However, caution will be advised when:

1. A 3-fold increase in LFTs above baseline for the patient is seen.

2. Any derangement in liver synthetic function is noted.

Intensive care unit staff (doctors or nurses) based in each clinical centre will identify potential participants. A log will be kept of patients meeting the inclusion criteria, describing the reasons if they are not subsequently recruited to the trial. The recruitment procedure to be followed is detailed in the SIGNET trial handbook (version 4 15-03-06).

### Informing potential participants about the trial

If the patient is able to give consent, ICU staff (doctors or nurses) will describe the study, backing oral information with the standard study information leaflet. If, as is more likely, the patient will be unable to give consent, intensive care unit staff will describe the study to relatives of the patient, backing oral information with the standard study information leaflet.

### Consent to participate

Once eligibility has been confirmed, authorised personnel will ask if the potential participant is ready to decide whether or not to join the trial. If so, she/he will give the participant a consent form. After the doctor or nurse has checked that the consent form is understood, the nurse or doctor will invite the participant to sign the form, then will add their own name and countersign it. In the case of relatives giving assent, the same procedure will be followed using the consent form. Copies of the signed consent form and information sheet will be placed in the hospital notes. If the participant subsequently becomes able to give consent, after assent has been obtained from the relative, the study will be described to the participant using the information sheet and consent form.

If the relative is not present and cannot attend the ICU to sign the written assent within 24 hours of the patient requiring PN, but is prepared to grant assent based on the information read out to them over the phone and subsequent discussion, verbal assent will be recorded. Written record of assent at a later time will be obtained if feasible. In all cases a written information sheet will be provided when they attend or a copy sent to them by post if they are unable to attend. Separate consent forms for verbal consent have been prepared.

In the unlikely event that there is no personal legal representative (next of kin) it is possible to recruit the patient to the study, provided a professional legal representative (or equivalent in England, Wales or Ireland) has been appointed by the hospital for that person.

### Information collected at trial entry

Once a participant has agreed to join the trial, ICU staff will record on a standard form:

#### 1. Identifying and contact information

• Full name, address, telephone number

• Date of birth, age on recruitment day, sex

• NHS, hospital number and Community Health Index number (if available)

• Marital status, woman's maiden name

• Name of a 'best contact', such as a friend or relative, with contact details

• General Practitioner's contact details

#### 2. Descriptive information

• Under-nourished, normal or obese, based on subjective clinical assessment [23]

• Patient groups – medical, or surgical (including trauma))

• APACHE (Acute Physiology and Chronic Health Evaluation) II score

• SOFA (Sequential Organ Failure Assessment) score

• Severe sepsis or septic shock

• Antibiotics/anti-infectives on recruitment

This information will be sent to the Trial Office.

### Trial interventions

The trial will use a 2 by 2 factorial design to test not only whether glutamine and selenium on their own are effective, but also whether there is extra effectiveness from the combination.

For this reason allocation will be to one of four groups, see table 1.

**Table 1:** The trial will use a 2 by 2 factorial design to test not only whether glutamine and selenium on their own are effective, but also whether there is extra effectiveness from the combination. For this reason allocation will be to one of four groups.

	**No glutamine**	**Glutamine**
**No additional selenium**	Standard PN bag 12.5 g nitrogen, 2000 kcal daily	PN bag, 12.5 g nitrogen (including 20.2 g glutamine), 2000 kcal daily
**Additional selenium**	Standard PN bag 12.5 g nitrogen, 2000 kcal daily 500 μg selenium daily	PN bag, 12.5 g nitrogen (including 20.2 g glutamine), 2000 kcal daily 500 μg selenium daily

All formulated PN bags (1500 ml volume) are isonitrogenous and isocaloric and have been tested for long-term stability by Fresenius-Kabi. Standard additions of fluid, electrolytes, vitamins and minerals will be allowed (including standard doses of selenium). dditional nitrogen and/or energy will not however, be allowed in the PN (to retain the isonitrogenous nature of the interventions). Supplementation will be given for seven days only (on ICU and subsequent wards and hospitals if practicable).

Once the decision is made to commence PN, it is desirable that SIGNET PN is given from the start. If this is not possible SIGNET PN should be started within 48 hours of commencing PN on ITU or HDU. If SIGNET PN is discontinued for any reason, e.g. line removed, SIGNET PN can be restarted within 48 hours from discontinuation and a total of 7 days of SIGNET PN may be given. Patients who have been given PN outside of critical care are eligible for recruitment. A patient may be recruited to the trial only if the pharmacy has sufficient stock for 5 days of bags of PN at the start of therapy.

The volume of feed may be reduced for men under 60 kg in weight and women under 70 kg. Consultants on intensive care may opt not to enter patients into the trial if they perceive the patient either to be at risk of refeeding syndrome, or to require a gradually phased introduction of nutrition support.

Patients entered in the trial will be monitored as is standard practice in the ICU.

### Treatment allocation

**Figure 1:**
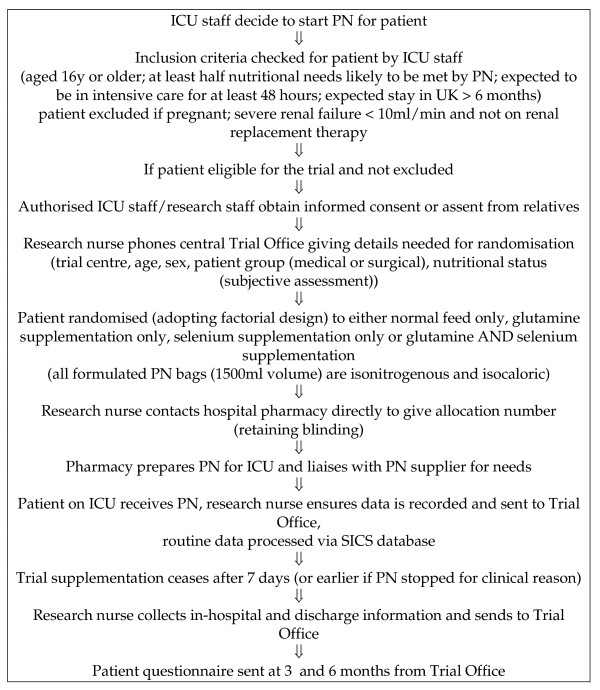
**Trial Flowsheet**. Randomised trial of glutamine and selenium supplemented parenteral nutrition for critically ill patients.

The existing central randomisation service (fully automated computerised telephone randomisation) in the Health Services Research Unit at the University of Aberdeen will be used to randomise patients. Recruiting staff will telephone the randomisation line and will be asked to key in details of identification and important prognostic factors (see below), when confirming eligibility and consent. Consent procedures will adhere to the 2003 Mental Health (Care and Treatment) (Scotland) Act. Random allocation to one of the four possible treatment combinations (see above) will be minimised on trial centre; age (<65 years or ≥ 65 years); sex; patient groups (medical or surgical (including trauma)); and, patient nutritional status (under-nourished, normal or obese – subjective clinical assessment [24]. A four digit number for that patient will be provided by the automated randomisation service, the research nurse accessing the randomisation service will then inform the centre pharmacy of this randomisation number. The trial office, based at the University of Aberdeen will have provided each pharmacy with a list of these random numbers linked to one of the four treatment allocations, thus maintaining the blinding of local investigators to the trial treatment. Figure 1 summarises these arrangements.

#### Subsequent arrangements

##### Informing key people

Following formal trial entry, the Trial Office or local research nurse will also contact:

• the ***consultant and ward staff ***caring for the patient on discharge from the intensive care unit – informing them of participation in the trial and the possible continuation of trial treatment.

• the ***general practitioner ***– informing the GP practice of participation in the trial and the (few) implications for the practice. This letter includes a brief description of the trial together with a request that the general practitioner notifies the Trial Office if the patient dies.

• the ***'best contact' ***asking for assistance to complete the questionnaires (see below)

### Additional information sought by post at three and six months after entry

Questionnaires will be sent to participants by post at three and six months post randomisation, which will contain:

i) The SF-12 questionnaire and the EQ-5D questionnaire

ii) Contacts with health services after discharge

A cost questionnaire will also be sent to participants by post at 6 months to elicit

(i) Costs to patients and carers (travel costs, time spent travelling and attending appointments, time lost from usual activities)

Participants will be sent up to two reminders by post if the questionnaires are not returned to the Trial Office.

If it is known that a participant is still in hospital at three and six months, questionnaires will not be sent to their home address. Instead the trial office will ask the study nurse or the staff looking after the participant to assist the participant in completing the questionnaires, if appropriate.

### Notifications by general practitioners

A letter and information sheet will ask the general practitioner to notify the Trial Office by telephone if the participant dies (and to give cause of death).

### Notifications by 'best contact'

A brief letter and freepost envelope will be sent to the 'best contact' (relative that gave assent) at the time of the participant's hospital discharge. The letter will ask if he/she could assist the participant to complete the three and six month questionnaires if this is appropriate.

### Role of study nurses following recruitment

#### Data collection

At least ten critical care units will be involved in the SIGNET trial. Potential trial sites listed in the ethics application are: Aberdeen Royal Infirmary, Western General Edinburgh, Royal Infirmary of Edinburgh, Western Infirmary Glasgow, Ninewells Hospital Dundee, Stirling Royal Infirmary, Dumfries and Galloway Royal Infirmary, Monklands Hospital Coatbridge, Southern General Hospital Glasgow, Victoria Infirmary Glasgow, Royal Alexandra Hospital Paisley, St John's Hospital Livingston, Queen Margaret Hospital Dunfermline, Raigmore Hospital Inverness. The SIGNET trial will start initially in the pilot sites, Aberdeen Royal Infirmary and the Western General Hospital in Edinburgh along with Ninewells Hospital, Dundee. The study may be extended to include sites outside Scotland if this is considered necessary in which case, possible sites include Cardiff Royal Infirmary, Belfast Royal Infirmary, and Newcastle Royal Infirmary.

The study nurse will ensure that data are fully collected for participants. The study nurse will also collect data needed for secondary care resource use (e.g. length of stay in ICU, HDU and general wards from hospital case notes.)

#### If the Trial Office fails to make contact

The Trial Office will liaise with the local study nurse if there is ever failure of contact with a participant. In these circumstances the nurse or Trial Office will telephone the general practitioner's (GP's) surgery to check vital status 24–48 hours before telephoning the participant. If the GP refuses to disclose this information, the patient will not be contacted.

#### Routine data collection

Information on subsequent hospital admissions and deaths will be collected by registration with the Information and Statistics Division Scotland (ISD) and the National Office of Statistics (ONS) and NHS Central registers

### Data collection and processing

Data from the various sources outlined above will be sent to the Trial Office in the University of Aberdeen for processing. Staff in Aberdeen will work closely with study nurses to secure as complete and accurate data as possible. A random 10% sample of data will be double entered to check accuracy. Extensive range and consistency checks will further enhance the quality of the data.

### Analysis plans

#### Ground rules for the statistical analyses

The statistical analyses will be based on all people randomised, irrespective of subsequent compliance with nutritional support. The principal comparisons will be:

(i) all those randomised glutamine versus all those not allocated glutamine

(ii) all those randomised selenium versus all those not allocated selenium

(iii) all randomised glutamine and selenium versus all randomised glutamine alone versus all randomised selenium alone versus all randomised placebo

Evidence will be sought for an interaction between glutamine and selenium.

#### Measures of outcome

##### Primary

• Mortality – on ICU and HDU and overall at six months

• Infections – counted as participants with new reported infections and Centres for Disease Control (CDC) confirmed infections in the first 14 days [25].

##### Secondary

• ICU and HDU length of stay and acute hospital length of stay

• Days of antibiotic usage;

• Duration of PN usage;

• Alive, ventilator-free days as recommended by Rubenfield [26];

• Change in SOFA scores;

• Serious Adverse events (excluding deaths) (for reporting of Suspected Serio-s Adverse Reactions (SSARs) and Suspected Unexpected Serious Adverse Reactions (SUSARs).

• Patient quality of life measured by SF12 and EQ5D; and

• Costs to the NHS, patients and carers/families; incremental cost per day in ICU saved and/or per quality adjusted life years (QALYs)

#### Timing and frequency of analyses

A single principal analysis is planned six months after the last person is recruited. If considered appropriate, follow-up of recruits will be extended at this time. The Data Monitoring Committee (DMC) (see below) will determine the frequency o- confidential interim analyses.

#### Secondary sub-group analyses

Planned subgroup analyses include:

(i) Under-nourished, normal or obese;

(ii) Patient groups (medical, or surgical (including trauma));

(iii) Age <65 or ≥ 65;

(iv) Evaluation by the severity of illness, including; APACHE II and SOFA scores, above and below mean or median score; and

(v) Severe sepsis/septic shock on recruitment

Stricter levels of statistical significance (2p < 0.01) will be sought, reflecting the exploratory nature of these subgroup analyses. The Chi-squared test for heterogeneity will be used to explore any apparent differential effects.

#### Economic analysis

The underlying aim is to keep economic data collection as parsimonious as possible to minimise the burden upon trial participants and participating centres. Data on costs to the NHS including treatments, investigations and consultations will be collected as part of the economic evaluation. The type of economic evaluation performed will depend upon the findings [23]. Both deterministic and stochastic sensitivity analysis [27] will be used to explore the importance of the uncertainty surrounding estimates of effectiveness, cost, and cost-effectiveness/utility. NHS resource use will be costed using appropriate unit costs. These will be taken from published sources (e.g. drug costs from the British National Formulary and GP cost from published estimates [28], manufacturers' list prices (e.g. consumables, equipment)) or using study-specific estimates. QALYs will be calculated using the EQ5D scores [29]. The EQ5D has been successfully used to measure changes in quality of life in patients discharged from ICU [30].

### Power calculation

The primary endpoint for the trial is the proportion of patients acquiring infection(s) after starting trial PN. Systematic reviews indicate that the reduction in proportion of patients acquiring infections in previous trials ranged from approximately 50% in the control arm to 40% in the intervention arm. Removing Griffiths' glutamine trial [31,32] – which only reported infections in a post-hoc analysis – shows the effect may indeed be greater (from 50% in the control arm to 30% in the intervention arm). A plausible reduction to detect would be the midpoint of these two estimates (i.e. a reduction from 50% to 35%), hence 340 participants would be required to detect this size of difference (with 80% power and 2p < 0.05) [33].

Given the high mortality rates in critical care patients [1], we have allowed for potential loss of patients to follow-up over the six months (see below – this would primarily affect secondary outcomes such as quality of life measures). We have consequently inflated the sample size requirement by approximately 20%, thus plan to recruit 500 patients to the trial.

Based on routine data available from the Scottish Intensive Care Society (SICS) audit database, the average length of ICU stay for patients receiving PN is approximately 9 (SD 7) days. For a 2 day reduction in ICU stay to be detected, with 80% power and 2p < 0.05, a trial of approximately 400 patients would be required. As we do not expect any dropout from the trial prior to discharge, we are adequately powered to detect this level of change.

A trial with 500 patients would allow us to detect a quarter of a standard deviation (0.25SD) change in hospital length of stay with 80% power and 5% significance (assuming all data for length of stay are available).

A trial of 340 patients would have 80% power and 5% significance to detect differences in Quality of Life measures of approximately 0.33SD (a third of a standard deviation change) or 90% power to detect a 0.5SD change. Previous research in the behavioural field suggests that a 0.5SD change is the level of change that should be associated with a moderate change in quality of life measures [34,35]. As such we are well powered to detect meaningful changes in quality of life measures.

#### Recruitment rates

Approximately 900 patients per annum receive PN in Scottish Intensive Care Units (source: SICS audit database). Assuming a conservative recruitment rate – that we recruit a third of eligible patients – we expect to recruit the 500 patients required within a two-year recruitment phase.

### Data analysis

Analysis of dichotomous outcomes will be analysed using statistical tests such the Chi-squared test. Continuous measures will be analysed using t-tests (with transformation of data to Normality if appropriate), or non-parametric equivalents. A further analysis accounting for the inter-relationship between deaths and length of stay will be undertaken, akin to that described for ventilator-free days by Rubenfield [26] mentioned earlier. This, for example, would allow the apparently short length of stays associated with early deaths to be accounted for appropriately. A significance level of 2p < 0.05 will be considered as evidence of statistical significance for the primary outcomes. Confidence intervals will also be presented where appropriate. The economic analysis will incorporate sensitivity analysis, and confidence intervals will be constructed around stochastic cost data using techniques such as bootstrapping [27].

### Organisation

#### Local organisation

Each collaborating centre will identify a principal investigator. The responsibilities of this person will be to:

1. establish the trial locally (for example, by getting agreement from clinical colleagues, facilitating local research ethics committee and trust approval; identifying and appointing a local study nurse; and ensuring that all clinical staff on ICU and the hospital pharmacy are aware of this trial)

2. agree to follow the trial protocol and to ensure that all team members on his/her site understand their obligations and to conduct the trial to GCP requirements

3. ensure that all study site personnel are adequately trained and qualified to carry out their delegated responsibilities and provide support and supervision to these personnel

4. agree not to start the trial until Main REC, LREC and local management approval is given

5. retain all documentation associated with the trial and all documentation sent by the Chief Investigator or Sponsor of the trial

6. report deviations from the protocol to the Chief Investigator

7. report any safety issues to the Chief Investigator

8. take responsibility for clinical aspects of the trial locally (for example, if any particular concerns emerge)

9. notify the Trial Office of any unexpected clinical events which might be related to trial participation

10. report serious adverse events to the Chief Investigator within 24 hours of the Principal Investigator knowing of the serious adverse events

11. provide support and supervision for the local study nurse

12. represent the centre at collaborator's meetings

Each clinical centre may appoint a ***study nurse ***to co-ordinate the day to day aspects of the trial. The responsibilities of this person will be to:

1. keep local staff informed of progress in the trial

2. keep regular contact with the local principal investigator, with notification of any problem or unexpected development

3. maintain regular contact with the Trial Office

4. identify potential participants and keep a log of whether or not they are recruited (with reasons for non-participation)

5. liaise with PN team

6. check eligibility, give information about the trial, and seek consent

7. randomise patients and obtain randomisation code

8. liaise with the hospital pharmacy

9. collect data describing participants, and send these to the Trial Office

10. liaise with consultants on other wards after patients have been discharged from ICU

11. record outcome measures on study CRFs

12. send study CRFs to Trial Office no more than eight weeks after each patient has been recruited

13. clarify the situation when the Trial Office fails to make a contact with a local participant, getting in touch by telephone

14. seek further clinical details when a major trial event (such as a hospital admission or death) is reported to the Trial Office, even if this occurs in another hospital

15. provide support for participants in other ways if there are difficulties

16. represent the centre at trial nurse meetings and collaborators meetings

Each clinical centre will designate ***study pharmacist(s) ***to co-ordinate the day-to-day pharmacy aspects of the trial. The responsibilities of the local pharmacists will be to:

1. allocate treatment for the patient according to the assigned randomisation code using the SIGNET randomisation schedule

2. ensure the allocation is checked by a pharmacist (other than ICU pharmacist if possible)

3. formulate the PN according to prescription, stability information and hospital SOPs

4. label bags appropriately to ensure blinding of treatment allocation is maintained at ward level

5. ensure that clinical trials pharmacists and aseptic pharmacists are the only personnel on site unblinded to treatment allocation

6. record the use of the SIGNET study PN bags and selenium if applicable on the accountability log

7. record the use of bags after every patient has completed their SIGNET PN on the pharmacy stock control website

8. ensure that all study site pharmacy personnel are adequately trained and qualified to carry out their delegated responsibilities and provide support and supervision to these personnel

9. manage stock levels to ensure sufficient stock of both glutamine and non glutamine bags to provide seven days of SIGNET PN when patient recruited

10. ensure that the storage conditions of the Signet Study PN bags are adhered to

11. ensure that there is stability data available for all variations in the electrolyte profiles of the Signet Study PN bags that are used

12. retain all documentation associated with the trial for example patient worksheets, completed prescriptions, study drug accountability logs, shipping receipt, destruction or disposal documentation, temperature control logs and all documentation sent by the Chief Investigator or Sponsor of the trial

13. report deviations from the protocol to the Principal Investigator

14. report any safety issues to the Principal Investigator

### Trial co-ordination

#### The Trial Office

The Trial Office is in the Centre for Healthcare Randomised Trial (ChaRT), in the University of Aberdeen, which gives day-to-day support to all clinical centres. It is responsible for collection of data (in collaboration with the local study nurses), data processing and analysis. It is also responsible for randomisation and unblinding, (as clinically necessary). The trial coordinator is based in Edinburgh and will supervise the day-to-day conduct of the trial under the direction of the Chief Investigator and the Director of CHaRT. The Trial Office will be responsible for submitting annual reports to MREC, MRC, and regular reporting of SSARs and SUSARs to MREC and MHRA. It will also produce progress reports to both the Trial Steering Committee, and unblinded interim reports to the Data Monitoring Committee (DMC).

There will be four staff directly located in the central Trial Office – the data manager/secretary, the statistician, the economist and the programmer. These staff members will be supported by senior members of CHaRT (The Director, the Senior Trial Manager and the Senior IT Manager and by the Director of HSRU and grantholder). The *trial co-ordinator *will be based in the University of Edinburgh but will be in day to day contact with the trial office and will be collocated in the trial office four days a month.

The *trial co-ordinator *will take overall responsibility for the day to day conduct of the trial, including supervision of all trial staff, visiting all centres and working with the local ICU investigators to establish the trial, providing regular liaison with the research nurses, arranging all trial meetings, and reporting to the project management group. The *data manager/secretary *will be responsible for all aspects of data collection and processing, including processing of trial entry data, data entry, questionnaire monitoring, chasing missing information, and follow-up of non-responses. The *programmer *will establish a database management system for efficient conduct of the trial including the randomisation, timely despatch of questionnaires, automatic form monitoring, data validation and cleaning. The *statistician *will undertake all statistical tasks in the study including the formal analysis and reporting of the data, progress reports to the Study Committees at study end, all under the supervision of the Director of HSRU (one of the grant holders).

A *health economist *will be responsible, under the supervision of the Senior Health Economist (one of the grant holders), for the development of data collection forms (such as the patient/carer time and travel questionnaire) required for the economic evaluation; the unit costs of health care resources; the analysis of the economic data; and the preparation of the economic evaluation component of the final report.

#### The Operations Management Group

The Operation Management Group will meet weekly at the Trial Office in Aberdeen, to review day to day progress and troubleshoot emerging issues. The Chief Investigator via teleconference will routinely join them.

#### The Project Management Group

This consists of representatives from two of the clinical centres, and representatives from the Trial Office. Observers may be invited to attend at the discretion of the Project Management Group. This will meet regularly during the trial – typically every 6–8 weeks face-to-face or via teleconference.

#### The Trial Steering Committee

A Trial Steering Committee supervises the trial. This committee includes three independent members, together with those originally granted funds to mount the trial. Observers from the host University (University of Edinburgh) and Medical Research Council may also attend. Other members of the Project Management Group may attend as observers at the invitation of the Chair of the Steering Committee.

#### Data and safety monitoring

##### The Data Monitoring Committee

A DMC will be established. This will be independent of the trial organisers. During the period of recruitment to the trial, interim analyses will be supplied, in strict confidence, to the DMC, together with any other analyses that the committee may request. This may include analyses of data from other comparable trials. In light of these interimfindings, the DMC will advise the Trial Steering Committee if one or more of the randomised comparisons in the trial has provided;

1. proof beyond reasonable doubt that for all or some types of patients, one particular type of treatment is clearly indicated in terms of a net reduction in participants with infection without any increased risk of death or serious complications (or clearly contraindicated because of a net increase in length of stay or mortality)

2. evidence that might reasonably be expected to influence materially the care of people who require PN feeding in ICU by clinicians who know the results of this and comparable trials.

The Trial Steering Committee can then decide whether or not to modify intake to the trial. Unless this happens, however, the Trial Steering Committee, project management group, clinical collaborators, and trial office staff (except the study statistician who supplies the confidential analyses) will remain blinded from the interim results.

The frequency of interim analyses will depend on the judgement of the chairman of DMC, in consultation with the Trial Steering Committee.

##### Other safety concerns

The incidence of adverse effects from the proposed dosage of glutamine and selenium is anticipated to be very low – in the systematic reviews safety issues were not a concern.

Collaborators and participants may write to the chairman of the Trial Steering Committee regarding any worries they may have about the trial. If concerns arise about side effects or particular types of participants, these will be relayed to the Chairman of the DMC.

If clinically indicated, rapid unblinding of trial materials will be available through the trial office.

The trial will be conducted according to the MRC Good Clinical Practice Guidelines (1998), the Data Protection Act 1998, and the ICH E6 GCP document.

### Publication

The success of the trial depends entirely on the wholehearted collaboration of a large number of participants, their relatives, nurses and doctors. For this reason, chief credit for the trial will be given, not to the committees or central organisers, but to all those who have wholeheartedly collaborated in the trial. The trial's publication policy is described in detail in section 2 of the Site File and is in accordance with the rules of the International Committee of Medical Journal Editors. The results of the trial will be reported first to trial collaborators. The main report will be drafted by the Project Management Group, and circulated to all clinical co-ordinators for comment. The final version will be agreed by the Trial Steering Committee before submission for publication, on behalf of the Collaboration. In this case the authorship will be presented by the collective title – The SIGNET Trial Group – and the article will carry a footnote of the names of the people (and their institutions) represented by the corporate title. For any additional publications (including conference presentations) one or more authors may take responsibility for drafting the paper or abstract but all group members qualify as members; in this case, this should be recognised using the byline 'Jane Doe *and *the Trial Group.' Group authorship may also be appropriate for publications where one or more authors take responsibility for a group, in which case the other group members are not authors but may be listed in the acknowledgement (the byline would read 'Jane Doe *for *the Trial Group'). No study or report can be published without the agreement of the Project Management Group.

Once the main report has been published, a summary will be sent to the GPs of participants involved in the trial and to individual participants' or relatives' who have indicated they would like to receive one.

## Discussion

In 2002, the Medicines Control Agency provided exemption letters for Selenase T pro injection (sodium selenite) supplied by Oxford Nutrition and for Dipeptiven supplied by Fresenius-Kabi for this trial. Prior to the implementation of the EU Directive in 2004, the MHRA confirmed that the exemptions would be rolled over to a Clinical Trial Authorisation.

The development of the protocol, ethical, research and development approvals had to negotiate the administrative constraints of the European Directive on Clinical Trials when applied to non-commercial trials.

Efficient and economical provision of parenteral nutrition at recruiting centres required collaboration with a pharmacy holding a license to compound the investigational medicinal product. We are grateful to Tayside Pharmaceuticals for their continued support and provision of 2 "base" bags of blinded parenteral nutrition, one with glutamine and one without. The two PN base bags have a 90 day stability. Thus, transportation and stock management are vital to the trial success and in particular, minimisation of wastage. Participating pharmacy departments and the central supplier have access to a custom designed website that has information on stock inventory, its shelf life and site PN usage. When this is combined with a comprehensive, refrigerated delivery service and an emergency contract with a national courier, PN supply has become more efficient.

Addition of selenium and electrolytes by the individual participating pharmacies allows SIGNET centres to recruit patients with only a 5 day stock of 2 "base" bags of PN. Debate exists as to whether the addition of selenium at each centre constitutes a licensable action. Notably, selenium is one of the 2 Investigational Medicinal Products (IMPs) for this trial. A final decision is expected from competent authority in due course.

Trial management is divided between Edinburgh where the Chief Investigator and Trial Coordinator are based and Aberdeen University where the trial office is based (as described above). The University of Edinburgh is administering the MRC Grant. SIGNET is a multicentre trial and the use of teleconferencing and the willingness of the current TrialCo-ordinator to travel has made the arrangement successful.

The EU clinical trials directive requests that each trial must have an identified sponsor. NHS Lothian is the lead NHS organisation and the Sponsor of the SIGNET trial with collaboration from the Centre for Healthcare Randomised Trials (CHaRT), within the University of Aberdeen. Increasing investment in trial supporting infrastructure has facilitated trial monitoring and documentation for site monitoring and source document verification. Pharmacovigilance is managed by Aberdeen University as the trial office has infrastructure and procedures in place for reporting Suspected Unexpected Serious Adverse Reactions (SUSARs).

The study is being conducted throughout Scotland and involves patients in intensive care and high dependency units. The collaboration and support of the staff in the Critical Care areas is essential in running this study. There are currently 10 sites in Scotland recruiting patients and to date over 285 patients have been recruited.

## Abbreviations

SIGNET Scottish Intensive care Glutamine or seleNium Evaluative Trial

PN Parenteral Nutrition

EN Enteral Nutrition

EU European Union

ICU Intensive Care Unit

CCU Critical Care Unit

UK United Kingdom

Dept Department

RCT Randomised Controlled Trial

CI Confidence Interval

RR Relative Risk

LFTs Liver Function Tests

NHS National Health Service

APACHE Acute Physiology and Chronic Health Evaluation

SOFA Sequential Organ failure Assessment

g gram

μg microgram

kcal kilocalorie

ml millilitres

Kg kilogram

SF-12 Short Form 12

EQ-5D Euoquol 5D [29]

ISD Information and Statistics Division (Scotland)

ONS National Office of Statistics

GP General Practitioner

SSAR Suspected Serious Adverse Reaction

SUSAR Suspected Unexpected Serious Adverse Reactions

QALYs quality adjusted life years

SICS Scottish Intensive Care Society

ChaRT Centre for Healthcare Randomised Trial

GCP Good clinical practise

LREC Local research ethics committee

REC Research ethics committee

CRF Case report form

SOP Standard operating procedure

MHRA Medicines and Healthcare products Regulatory Agency

DMC Data monitoring committee

TSC Trial steering committee

MRC Medical Research Council

## Competing interests

Peter JD Andrews (Chief Investigator), Alison Avenell, Claire Battison, Marion K Campbell, Bernard L Croal, Anne C Milne, David Noble, John Norrie, William G Simpson, Luke D Vale, Jonathon Cook & Robyn de Verteuil – The authors declare that they have no competing interests.

## Authors' contributions

DN, AA, PJDA, MC, LV, BC, WS conceived the study, participated in its design and drafted the manuscript. JN, JC, RV participated in the design. All authors have read and approved the final manuscript.
